# Diagnosis, Clinical Features and Management of Interstitial Lung Diseases in Rheumatic Disorders: Still a Long Journey

**DOI:** 10.3390/jcm11020410

**Published:** 2022-01-14

**Authors:** Marco Sebastiani, Caterina Vacchi, Giulia Cassone, Andreina Manfredi

**Affiliations:** 1 Rheumatology Unit, University of Modena and Reggio Emilia, Azienda Ospedaliero-Universitaria Policlinico di Modena, 41124 Modena, Italy; caterina.vacchi@unimore.it (C.V.); giulia.cassone@unimore.it (G.C.); andreina.manfredi@gmail.com (A.M.); 2 Clinical and Experimental Medicine PhD Program, University of Modena and Reggio Emilia, 41124 Modena, Italy

Interstitial lung disease (ILD) is one of the most frequent pulmonary complications of autoimmune rheumatic diseases (ARDs), and it is mainly associated with connective tissue diseases (CTDs) and rheumatoid arthritis (RA) [1,2].

Lung involvement is very common in ARDs, and it can include a wide spectrum of disorders, possibly involving airways, pleura, parenchyma and vascular tree bronchiectasis, nodules, and it can cause issues ranging from infections to drug toxicity. In many patients, different types of lung involvement can be observed at the same time, causing heterogeneous clinical pictures in the same patient at different times in their clinical history [3].

The severity and clinical course of ILD is largely variable, but it generally represents one of the main causes of morbidity and mortality in patients with ARDs [1].

The prevalence of ILD reported in patients with CTDs (CTD-ILD) widely differs according to the different classification modalities among case series and registry studies and among the specific rheumatologic diagnoses, as the prevalence is higher in patients with systemic sclerosis (SSc) and idiopathic inflammatory myopathies (IIM) and lower in those with systemic lupous erythematosus [3]. Recently, the European Respiratory Society (ERS) and the American Thoracic Society (ATS) “Task Force on Undifferentiated Forms of Connective Tissue Disease-associated Interstitial Lung Disease” proposed classification criteria for a new research category defined as “Interstitial Pneumonia with Autoimmune Features” (IPAF), including a subgroup of patients with ILD and clinical, radiological and/or serological findings suggestive of but not diagnostic of a definite CTD, but this remains to be better investigated [4].

While for SSc and IIM the routine diagnostic work-up always includes the study of lung involvement, the search for ILD is not usually performed in other rheumatic diseases, such as RA, primary Sjogren’s syndrome (pSS) and systemic lupus erythematosus (SLE). Therefore, we have a lack of knowledge on the epidemiology and clinical history of ILD with regard to these latter diseases; the knowledge that we do have is mainly taken from in retrospective studies that provide no conclusive data [5].

For example, despite the fact that RA is the most common inflammatory arthropathy, the prevalence and characteristics of lung involvement are largely unknown. In fact, in previous studies, the prevalence of RA-ILD largely varied according to the heterogeneity of the populations, ILD definitions, as well as the diagnostic tools employed in the studies [6].

Although clinically evident ILD is usually recorded in 7–10% of RA patients, the prevalence of subclinical ILD was higher when consecutive patients were evaluated using high-resolution computed tomography (HRCT). In this regard, Dawson identified 19% of 150 consecutive RA patients, of which only 54% were shown to have low-grade dyspnea and 14% were shown to have restrictive syndrome during lung function tests (LFTs) [7]; Gochuico observed a “preclinical” ILD using HRCT in about one third of the study population, without dyspnea or cough but characterized by a long-standing joint disease [8]; finally, Gabbay identified abnormalities compatible with ILD in 33% of patients with a disease onset of RA of less than 2 years [9]. In an ongoing prospective study evaluating the epidemiology of RA-ILD (LIRA study, Lung Involvement in Rheumatoid Arthritis), preliminary data showed a prevalence of 20.1% [10].

Moreover, only in recent years has it been observed that ILD can precede the diagnosis of ARDS in many cases; in particular, many cohorts of non-sicca onset pSS have been described. In a recent study on patients with pSS-ILD, half of 34 patients were firstly referred to a pulmonologist because of respiratory symptoms or the occasional diagnosis of ILD [11]. Moreover, Hyldgaard and co-workers reported that fourteen per cent of ILD cases were diagnosed 1–5 years before RA, while 34% were diagnosed in a period ranging from 1 year prior to after the RA diagnosis [2].

Finally, we recently published a study made up of a cohort of 58 patients with positivity for anti-myeloperoxidase (MPO) antibodies, in which a rheumatic disease was developed in more than 20% of cases during a median follow-up of 39 months, mainly ANCA-associated vasculitis [12].

The diagnosis of lung involvement in ARDs patients on the basis of respiratory symptoms alone can be difficult: patients with mild or slowly progressive lung disease can be asymptomatic for a long period of their clinical history, and some suggestive clinical manifestations, such as fatigue, dyspnea and cough, can also derive from extra-thoracic causes, such as arthritis, muscle involvement and sicca syndrome [6]. Currently, HRCT remains the gold standard for diagnosis, and it is mandatory in the case of suspected ILD. In fact, chest X-ray is considered insensitive in identifying ILD and it should not be used in screenings of ILD; chest X-ray retains a role in identifying pulmonary complications, such as pleural effusion, infection or lung cancer [13].

Nevertheless, a routine use of HRCT for screening programs can only be proposed in diseases with high prevalence of ILD, such as antisynthetase syndrome or systemic sclerosis, while in other conditions with lower incidences of ILD, a screening with HRCT is not advisable due to both high cost and X-ray exposure [14,15].

Therefore, rheumatologists and pulmonologists are in search of simple and repeatable tools for the screening of ILD in patients with ARDs. Although it may be unremarkable in some cases, physical lung examination has been proposed as an easy screening method for the early diagnosis of interstitial pneumonia, both idiopathic and secondary to other diseases [16]. In fact, lung auscultation can reveal fine bibasilar, end-inspiratory, ‘Velcro-like’ crackles, which may precede the development of clinically overt ILD [16]. Recently, we proposed an algorithm named VECTOR (VElcro Crackles detecTOR) that is able to recognize Velcro crackles in pulmonary sounds from RA and CTDs patients with a diagnostic accuracy of about 80% [15,16].

In recent years, lung ultrasound examination has been proposed as a useful, feasible, non-invasive imaging technique with high sensitivity and specificity for the screening and monitoring of ILD in patients affected by CTDs, mainly systemic sclerosis [17]. In other ARDS, such as RA or pSS, published data are scarce, and lung ultrasound reproducibility and usefulness have not been established yet [18].

In patients with ILD, an LFT may detect a restrictive ventilatory failure, characterized by reduced forced vital capacity (FVC), increased forced expiratory volume in 1 s (FEV1) and a normal FEV1/FVC ratio, together with a decreased diffusing capacity of the lung for carbon monoxide (DLCO), even in the absence of symptoms [19]. However, an LFT is useful for monitoring the evolution of ILD, but since its sensitivity in early stages of the disease is quite low, an LFT cannot be proposed as a screening method for the diagnosis of ILD.

Sometimes, ILD and airway disease may be concomitant, for example, in combined pulmonary fibrosis and emphysema; in these cases, spirometry can be normal, and only a reduction in DLCO may occur [20]. As DLCO is highly sensitive in detecting ILD, in early involvement, LFT impairment may only be characterized by DLCO reduction, whereas FVC may be more useful for the assessment of disease extension [19]. In patients with a disproportion between DLCO and FVC, with a reduction in DLCO and the stability of FVC, pulmonary arterial hypertension should be suspected independently by the presence and severity of ILD [19].

FVC is highly reproducible, and its changes are specific to ILD once extrapulmonary restriction due to pleural or muscle diseases has been excluded. Disease progression may be expected when a decline of at least 10% in FVC or of at least 15% in DLCO over 6–12 months is recorded [21].

After diagnosis, the management of ILD associated with ARDs is quite complex because of the heterogeneity of clinical history alone and in combination with the possible systemic manifestations of ARDs. So, the management and the treatment of these patients must always be evaluated with a multidisciplinary approach, including a pulmonologist, radiologist and rheumatologist.

In any case, a close collaboration between a rheumatologist and pulmonologist is required in at least three situations:

- Differential diagnosis between idiopathic interstitial pneumonia and ILD related to ARDs. Nowadays, it is quite clear that ILD can precede the occurrence of joint or systemic manifestations in ARD patients, and the involvement of a rheumatologist can also facilitate the correct diagnostic definition in patients with a UIP pattern and absent or sub-clinic systemic involvement. The role of a rheumatologist in MDD, first proposed to increase the diagnostic accuracy of IPF, has not been widely investigated [22]. In 2018, Levi prospectively evaluated the effect of the inclusion of a rheumatologist in multidisciplinary discussions (MDDs) for the assessment of ILD diagnosis, concluding that this approach could significantly increase diagnostic accuracy and reduce the number of invasive procedures that are performed [23]. This result has recently been confirmed by a systematic review; despite the fact that it was not conclusive, the review highlighted that the participation of a rheumatologist in diagnostic MDDs reduced the risk of the misclassification of ILD patients [24].
- The management of lung involvement. The interpretation of lung function, the evaluation of radiologic ILD patterns and the evaluation of lung disease progression should be always evaluated together by a pulmonologist and radiologist. Although variable, the course of ILD may result in respiratory failure, mainly in progressive fibrosing diseases. While disease course is well described for idiopathic pulmonary fibrosis (IPF), characterized by a UIP pattern, it is not so well defined in ILD associated with ARDs. However, a progressive fibrosing disease has been also described: interstitial pneumonia secondary to rheumatic diseases, mainly RA and SSc [25]. Recently, a progressive fibrosing pattern has been reported that also has a high prevalence in patients with pSS [26]. According to the results of the INBUILD trial on the efficacy of nintedanib in fibrotic ILD different from IPF, antifibrotic treatment could also be effective in patients with ARDs and progressive fibrosing ILD [25]. If these results can be confirmed in larger populations of patients with ILD associated with ARDs, antifibrotic drugs, namely nintedanib and pirfenidone, could represent an important therapeutic option for these patients, alone or in combination with immunosuppressive drugs.
- The management of acute complications of lung involvement in patients with ARDs. Infections and acute exacerbation (AE) are frequent, severe complications of ILD, including ARDs-related ILD. Moreover, the differential diagnosis between drug-induced, acute ILD and AE of ARDs-related ILD is difficult and often based on the temporal relationship between drug initiation and the development of symptoms and/or on improvement upon drug discontinuation. The early diagnosis of a severe complication such as AE-ILD and the right referral to highly specialized centers are essential for increasing the survival of these patients [27].

Moreover, RA patients often present many cardiovascular and respiratory comorbidities other than ILD. In these cases, MDDs increase the ability of a rheumatologist to evaluate LFT, clinical and radiological data, as well as during follow-up for the assessment of disease severity and progression. Patients with RA typically undergo immunosuppressive treatment with potentially increased infectious risk; when these occur, MDDs can become mandatory to discriminate between infection and disease progression [6].

Another situation in which an MDD is essential is the definition of the radiologic and/or histologic pattern of ILD. The relevance of ILD pattern is different in CTDs, such as systemic sclerosis, compared to in RA. In fact, more studies showed that the presence of a UIP pattern did not worsen the prognoses of patients with SSc [28], while the survival rates of RA-ILD patients with a UIP pattern seemed to be similar to that of patients with IPF [6]. However, we need larger prospective studies to confirm these data and to investigate the role of UIP patterns in other conditions, namely pSS, in which evidence-based data are still lacking.

The clinical history, treatment and prognosis of patients with ILD associated with ARDS remains largely unknown.

There are no therapeutic recommendations for the treatment of ILD related to ARDS. Therapeutic options for RA-ILD are complicated by the possible pulmonary toxicity of many disease-modifying anti-rheumatic drugs (DMARDs) and by their unclear efficacy on pulmonary disease. Therefore, joint and lung involvement should be evaluated independently of each other for treatment purposes. In general, treatment strategies are based on the use of steroids and immunosuppressive drugs, both for steroid-sparing purposes and for those patients who do not respond, but there are currently no reliable data on the efficacy of these therapies [6,29,30,31,32].

Only prospective studies, possibly including homogeneous populations of patients with specific rheumatic diseases, can help to increase our awareness of this severe complication; in the meantime, the only possible approach to the multifaceted aspects of these patients is the close collaboration between expert physicians from many different specialties, with the most important being rheumatology, pulmonology and cardiology experts.
